# Small changes in bone structure of female α7 nicotinic acetylcholine receptor knockout mice

**DOI:** 10.1186/s12891-015-0459-8

**Published:** 2015-01-31

**Authors:** Katrin S Lips, Özcan Yanko, Mathias Kneffel, Imke Panzer, Vivien Kauschke, Maria Madzharova, Anja Henss, Peter Schmitz, Marcus Rohnke, Tobias Bäuerle, Yifei Liu, Marian Kampschulte, Alexander C Langheinrich, Lutz Dürselen, Anita Ignatius, Christian Heiss, Reinhard Schnettler, Olaf Kilian

**Affiliations:** Laboratory for Experimental Trauma Surgery, Justus-Liebig University Giessen, Kerkraderstr. 9, 35394 Giessen, Germany; Institute for Physical Chemistry, Justus-Liebig University Giessen, Heinrich-Buff-Ring 58, 35392 Giessen, Germany; Institute of Radiology, University Medical Center Erlangen, Friedrich-Alexander University Erlangen-Nuernberg, Palmsanlage 5, 91054 Erlangen, Germany; Department of Medical Physics in Radiology, German Cancer Research Center, INF 280, D-69120 Heidelberg, Germany; Department of Radiology, Justus-Liebig University Giessen, Schubertstr. 81, 35392 Giessen, Germany; Department of Diagnostic and Interventional Radiology, BG Trauma Hospital, Friedberger Landstraße 430, 60389 Frankfurt/Main, Germany; Institute of Orthopaedic Research and Biomechanics, Center of Musculoskeletal Research University of Ulm, Ulm, Germany; Department of Trauma Surgery Giessen, University Hospital of Giessen-Marburg, Justus-Liebig University Giessen, Rudolf-Buchheim-Str. 7, 35392 Giessen, Germany; Department of Orthopedics and Trauma, Zentralklinik Bad Berka, Robert-Koch-Allee 9, 99437 Bad Berka, Germany

**Keywords:** Nicotinic receptor, Bone strength, Bending stiffness, Cathepsin K, ToF-SIMS, Osteoid, Knockout mice, Micro-CT

## Abstract

**Background:**

Recently, analysis of bone from knockout mice identified muscarinic acetylcholine receptor subtype M3 (mAChR M3) and nicotinic acetylcholine receptor (nAChR) subunit α2 as positive regulator of bone mass accrual whereas of male mice deficient for α7-nAChR (α7KO) did not reveal impact in regulation of bone remodeling. Since female sex hormones are involved in fair coordination of osteoblast bone formation and osteoclast bone degradation we assigned the current study to analyze bone strength, composition and microarchitecture of female α7KO compared to their corresponding wild-type mice (α7WT).

**Methods:**

Vertebrae and long bones of female 16-week-old α7KO (n = 10) and α7WT (n = 8) were extracted and analyzed by means of histological, radiological, biomechanical, cell- and molecular methods as well as time of flight secondary ion mass spectrometry (ToF-SIMS) and transmission electron microscopy (TEM).

**Results:**

Bone of female α7KO revealed a significant increase in bending stiffness (p < 0.05) and cortical thickness (p < 0.05) compared to α7WT, whereas gene expression of osteoclast marker cathepsin K was declined. ToF-SIMS analysis detected a decrease in trabecular calcium content and an increase in C_4_H_6_N^+^ (p < 0.05) and C_4_H_8_N^+^ (p < 0.001) collagen fragments whereas a loss of osteoid was found by means of TEM.

**Conclusions:**

Our results on female α7KO bone identified differences in bone strength and composition. In addition, we could demonstrate that α7-nAChRs are involved in regulation of bone remodelling. In contrast to mAChR M3 and nAChR subunit α2 the α7-nAChR favours reduction of bone strength thereby showing similar effects as α7β2-nAChR in male mice. nAChR are able to form heteropentameric receptors containing α- and β-subunits as well as the subunits α7 can be arranged as homopentameric cation channel. The different effects of homopentameric and heteropentameric α7-nAChR on bone need to be analysed in future studies as well as gender effects of cholinergic receptors on bone homeostasis.

## Background

Acetylcholine acts as a neuronal as well as a non-neuronal signaling molecule through binding to nicotinic (nAChR) and muscarinic acetylcholine receptor (mAChR). nAChR are ligand gated cation channels build up by 5 subunits [1]. The composition of subunits in nAChRs determines ligand specificity, ligand affinity, cation permeability, and channel kinetics [2]. nAChR formed by α1, β1, γ, δ and ε subunits are called the muscle type of nAChR that is particularly localized in the skeletal motor unit. The neuronal type of nAChR is present in the central nervous system and non-neuronal cells. nAChR are built up as heteropentamers by α- and β-subunits (α2-7, β2-4), as homopentamers by α-subunits (α7, α9) and as α-heteropentamers by different α-subunits (α9α10, α7α10) [3,4]. Bajayo et al. reported that mice deficient of the nAChR subunit α2 have increased bone resorption and low bone mass [5]. Several nAChR subunits and mAChR occur in bone tissue [6-8]. Besides nAChR subunit α2 mAChR are also involved in bone mass regulation. Activation of mAChR subtype M3 (M3R) leads to an increase in bone biomechanics, collagen synthesis, formation of trabeculae [9,10] as well as a decrease in bone resorption [9]. Thus, the mAChR M3 as well as the nAChR subunit α2 has been identified as positive regulator of bone mass accrual. In our previous study we compared the bone of male mice deficient for α7-nAChR (α7KO) compared to their corresponding wild-type mice (α7WT) where we did not find significant differences [10]. Since alterations in the collagen expression in the skin of α7KO [11] were demonstrated and regulations of bone mass is most prominent in females (e.g. osteoporosis) [12] we decided to conduct a study where the bone of female α7KO is analyzed. In addition to the gender change we also included some complementary methods. One of them was “time of flight secondary ion mass spectrometry” (ToF-SIMS) for quantification of bone calcium ion (Ca^2+^) content and proline fragments that is one of the main amino acids of collagen [13-16]. The principle of ToF-SIMS is that primary ions hit the sample surface, releasing secondary ions from the surface, that were collected by an analyzer for producing single mass spectra and mass images of the sample surface [14]. Besides ToF-SIMS, several cell and molecular biological methods were used to analyze the bone microstructure and strength for which we could determine an increase in bone mass in female α7KO.

## Methods

### Animals

Female Chrna7 knockout mice (originally described by Orr-Urtreger et al. [17] on a C57BL/6 J background were derived from heterozygous breeding of animals obtained from Jacksons Laboratories (Bar Habor, ME, USA). Female 16 weeks old non-transgenic (α7WT, n = 8) and homozygous null mice (α7KO, n = 10) from this cross were used in this study. Animal care and experimental procedures were performed in accordance to the Directive 2010/63/EU and approved by the local animal care committee (Regierungspräsidium Gießen V 54–19 c 20–15 (1) GI 20/28 Nr. 88/2009). The animals were kept under specified pathogen-free conditions in the local animal breeding facility under a 12 hour (h) light–dark cycle with free access to water and chow.

Mice were genotyped by PCR described earlier [18]. In brief DNA was extracted from tail snips using the DNeasy Kit (Qiagen, Hilden, Germany). PCR was performed using gene-specific primer pairs (α7WT- and α7KO-forward primer: 5′-CCTGGTCCTGCTGTGTTAAACTGCTTC-3′, α7WT reverse primer: 5′-CTGCTGGGAAATCCTAGGCACACTTGAG-3′, α7KO reverse primer: 5′-GACAAGACCGGCTTCCATCC-3′) and the AmpliTaq Gold polymerase (Applied Biosystems, Branchburg, NJ, USA).

### Dual-energy X-ray absorptiometry (DXA)

Bone mineral density (BMD; g/cm^2^) of left femur, spinal column, and the whole skeleton were measured directly after euthanasia of the animals by DXA scan (lunar prodigy, GE Healthcare, Germany). Analysis was performed using the small-animal mode of the encore software (GE Healthcare, version 13.40) and was calibrated at each start of the experiment.

### 3D Micro-CT

The right femora, vertebrae Th13 and L1 were scanned with a high-resolution microtomographic system (Micro-CT 1072, SkyScan, Kontich, Belgium) at 16 μm voxel size at 8 bit gray scale range as described earlier [10]. The three-dimensional microstructural properties of the trabecular bone region of interest in the metaphysis and the vertebral body were assessed by using the SkyScan-CT-analyser software (SkyScan). The following parameters were calculated: bone volume fraction (BV/TV), trabecular thickness (Tb.Th), trabecular separation (Tb.Sp), trabecular number (Tb.N), structure model index (SMI), and bone surface density (BS/TV). In addition, the average cortical thickness was calculated. Mid-diaphyseal cortical area (Ct.Ar), relative cortical bone area to tissue area (CT.Ar./Tt.Ar), and average cortical thickness (Ct.Th) were obtained from a stack of 200 axial slices of the right femur.

### Biomechanical analysis of femoral bones

The mechanical properties of the diaphysis of femoral bones were evaluated by three-point bending test as described earlier [10,19]. In brief, the proximal femur was secured in a hinge joint after potting in dental cement. The distal end was positioned with the femoral condyles on a cylindrical support. Subsequently the bending load was introduced midway between both femoral ends until failure occurred. Bending stiffness (S in Nmm^2^), maximal strain (σ_u_ in MPa) and elasticity modulus (E in GPa) were calculated using the linear slope of the load-deformation curve.

### Dynamic contrast-enhanced magnetic resonance imaging (DCE-MRI)

MRI was performed on a 1.5 T MR-scanner (Symphony, Siemens, Germany) using an appropriate home-built coil for radiofrequency excitation and detection. The animals were imaged using a saturation recovery turbo flash sequence for dynamic contrast-enhanced imaging (orientation axial, TR 13 ms, TE 5.3 ms, Ti 300 ms, slice thickness 2 mm, TOV 60 × 60 mm^2^, matrix 128, measuring time 14:55 min) while infusing 0.1 mmol/kg Gd-DTPA (Magnevist, Schering) i.v. over a time period of 10 seconds. The vascular parameters amplitude A [arbitrary units], associated with relative blood volume) and exchange rate constant Kep ([1/min], reflecting vessel permeability) were calculated from DCE-MRI data according to the pharmacokinetic two-compartment model of Brix et al. [20]. Analysis was done using Dynalab workstation (Mevis Research, Bremen, Germany) drawing regions of interest (ROIs) manually in the lumbar L5 vertebrae, pelvis and femur.

### Histology, transmission electron microscopy (TEM)

Right tibia and vertebrae L3 were fixed in 4% phosphate-buffered paraformaldehyde (PFA, Merck, Darmstadt, Germany), decalcified with 10% EDTA, dehydrated in an increasing series of ethanol, embedded in paraffin and cut with a rotations microtome (Leica RM2155, Wetzlar, Germany) in 3–5 μm thick sections. Sections were processed for standard hematoxylin-eosin staining, enzyme- and immunohistochemistry.

Sections of vertebrae L4 were used for histology and transmission electron microscopy (TEM). Samples were fixed in yellow fix (2% paraformaldehyde, 2% glutaraldehyde, 0.02% picric acid) for 6 hours (h), washed several times with 0.1 M cacodylate buffer (pH 7.2), incubated in 1% osmium tetroxide (OsO_4_, Fluka, Buchs, Switzerland) and dehydrated in a series of graded ethanol. Subsequently, the samples were embedded in Epon (Serva, Heidelberg, Germany) and cut with an ultracut (Reichert-Jung, Germany). Semithin sections with a thickness of 0.5-1 μm were used for histology and ToF-SIMS analysis. For ultrastructural evaluations sections were cut at a thickness of 70–80 nm, counterstained with uranyl acetate and lead citrate (Reichert Ultrastainer, Leica, Germany), and examined in a Zeiss EM 109 transmission electron microscope and a digital camera (2 K, Zeiss, Oberkochen, Germany).

### Enzyme- and immunohistochemistry

Deparaffinized sections of L3 were used for enzyme histochemical staining of tartrate-resistant acidic phosphatase (TRAP) to identify osteoclasts and macrophages. In brief, sections were preincubated in sodium acetate buffer (pH 5.2) for 10 min and then incubated for 30 min at 37°C in a solution of Naphtol AS-TR phosphate (Sigma-Aldrich, Taufkirchen, Germany), NN-Dimethylformamid (Sigma-Aldrich), and sodium tartrat (Merck, Darmstadt, Germany) with fast red TR-salt (Sigma-Aldrich). Sections not used for histomorphometrical analysis were counterstained with hematoxylin (Shandon Scientific Ltd, Cheshire, UK) and coverslipped with Kaisers Glyceringelatine (Merck).

For immunohistochemistry deparaffinized sections were preincubated for 5 min in 3% H_2_O_2_ in Tris–HCl buffer (pH 7.4) with 5% Triton-X-100 (TBS) to block the endogenous peroxidase. After carefully washing the following primary antibodies were processed overnight in dilution buffer (Dako, Glostrup, Denmark) at 4°C: a) collagen type 1 (1:500 diluted, Biomex, Mannheim, Germany), b) PECAM-1 (CD31, 1:100 diluted, Acris, Herford, Germany), and c) smooth muscle actin (SMA, 1:100 diluted; Acris). After rinsing sections were incubated with goat anti-rabbit secondary antibody (1:500; Vector, Burlingame, California, USA) for 30 min and afterwards with the ABC complex/horseradish peroxidase labeled avidin (Dako) also for 30 min. Peroxdiase activity was visualized with Nova Red (Vector). Counterstaining of nuclei was performed with hematoxylin.

### Histomorphometric analysis

TRAP stained sections of vertebrae L3 were digitalized with an Axioplan-2 Microscope (Zeiss, Jena, Germany) equipped with a DC500 camera (Leica, Wetzlar, Germany) and analyzed with the open-source software TRAP Histo [21]. According to [22] the relative osteoclast parameter a) osteoclast surface/bone surface (Oc.S/BS), b) osteoclast number/bone surface (N.Oc/BS), c) osteoclast number/bone volume (N.Oc/BV), and d) osteoclast number/tissue volume (N.Oc/TV) were calculated.

### Real-time reverse transcriptase polymerase chain reaction (real-time RT-PCR)

Vertebrae L3 were transferred into RNAlater® (Applied Biosystems/Ambion, Austin, USA) directly after euthanasia. RNA isolation, removal of genomic DNA contaminations, cDNA synthesis as well as real-time RT-PCR with gene specific primers (Table 1, MWG, Ebersberg, Germany) were performed as described earlier [10].

**Table 1:** **Primer pairs used for real**-**time RT**-**PCR**

**Primer**	**Sequence**	**Length** **[bp]**	**Annealing temp.** **[°C]**	**GenBank ID** **(accession)**
Col1α1^a^ for	TGCCTGGCGGACTTCCTGGT	144	62	NM_007742.3
rev	CCGGCACCTGGCTTAGGTGG			
Col1α2^b^ for	ATGCCGGTCGACCTGGGGAA	144	60	NM_007743.2
rev	GTCCAGCTGGGCCGATTGGG			
CtsK^c^ for	GAGGCGGCTATATGACCACT	119	58	NM_007802.3
rev	CTTTGCCGTGGCGTTATACA			
Bglap^d^ for	TTCTGCTCACTCTGCTGACC	111	58	NM_007541.2
rev	TATTGCCCTCCTGCTTGGAC			
ALP^e^ for	TTAAGGGCCAGCTACACCAC	150	58	NM_007431.2
rev	CCTTCACGCCACACAAGTAG			
βActin for	TGTTACCAACTGGGACGACA	165	58	NM_007393.3
rev	GGGGTGTTGAAGGTCTCAAA			

^a^Col1α1: Collagen 1α1, ^b^Col1α2: Collagen 1α2, ^c^CtsK: Cathepsin K, ^d^Bglap: Osteocalcin, ^e^ALP: alkaline phosphatase.

### ToF-SIMS

The ToF-SIMS measurements were done with a TOF.SIMS 5–100 (IONTOF GmbH, Münster, Germany). The machine was equipped with a 25 keV Bismuth cluster primary ion gun. Data evaluation is done with the software Surface Lab 6.2. For the measurement of qualitative mass images Bi^+^ is used as primary ion species. The primary ion gun worked in the burst alignment (bi-ba-i) mode to obtain a lateral resolution of up to 300 nm. The measured area covered 250 × 250 μm^2^ with a pixel number of 512 × 512 pixel. For each mass image 150 scans were added.

For the comparison of the trabecular composition Bi_3_^+^ was used as primary ion species. The gun worked in the high current bunched (bi-hc-bu) mode. Due to the comparably high sample charging always an area of 500 × 500 μm^2^ with 128 × 128 pixel was measured. In each case 100 scans were added to obtain a better contrast. From each sample we took mass images from three different areas. For getting a good statistic we defined on each mass image three so called regions of interest (ROI) of the size 20 × 20 μm^2^ on the trabecular network. Only the mass spectra from these areas were summed up and evaluated. By this method we got 9 data evaluation areas per sample. In total we investigated 6 samples of wild type mice and 6 samples of knockout mice.

For the detailed measurement of the trabecular network a higher magnification is necessary. Therefore we operated the primary ion gun in the burst alignment mode and used Bi^+^ primary ions for getting higher primary ion currents. For the comparison of the composition of the trabecular edge with the center we measured areas of 31.3 × 31.3 μm with a pixel number of 128 × 128 pixel 50 times and defined again ROIs, which are located at the edge and in center of the trabeculae. Again the mass spectra of these ROIs were added and evaluated. From each sample we took two mass images and defined in each mass image two ROIs at the edge and two ROIs in the center of the trabeculae.

These semi-quantitative evaluations are based on the fact that the count rates of mass signals are proportional to the real concentrations of the respective species.

### Statistical analysis

The SPSS software (version 18.0; SPSS Institute Inc, Cicago, USA) was used for statistical analysis of real-time RT-PCR, histomorphometry, Micro-CT, DXA, and ToF-SIMS. Comparisons were performed by Kruskal-Wallis non-paramteric analysis of variance followed by the Mann–Whitney test. For statistical analyses of DCE-MRI, means and medians of parameters A and Kep were calculated for each region per group (threshold was set to be 1.5 [arbitrary units] for parameter A and 100 [1/min] for parameter Kep). Statistical comparison between the groups was performed for all ROIs using the t test (SigmaStat 3.5, Systat SigmaPlot v11.1, Systat Software Inc.). A p value of less than 0.05 was taken as a significant difference.

## Results

### Bone strength

**Figure 1:**
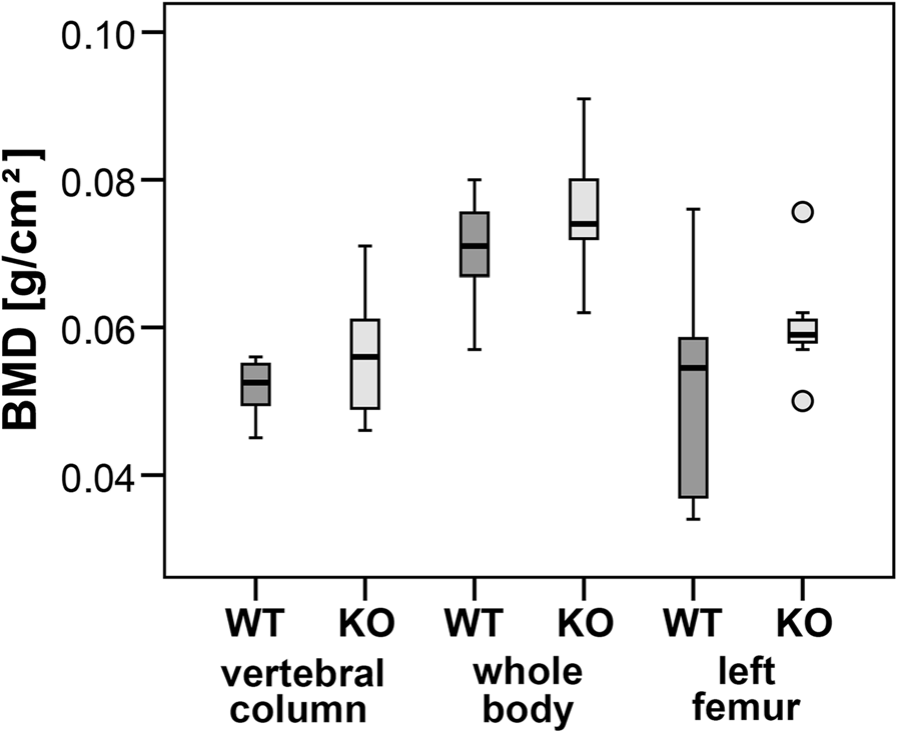
**Bone mineral density** **(BMD).** BMD of vertebral column, whole body and left femur was determined by DXA measurement of α7KO (KO) and α7WT (WT). Data presented as box plots with the median indicated by solid line within the box, percentile 25 and 75 as bottom and top of the box, and percentile 0 and 100 as whiskers. Small circles represent data beyond 3x SD.

**Figure 2:**
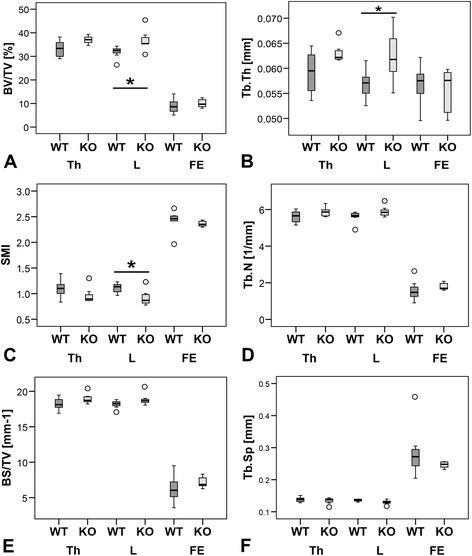
**Bone microarchitecture.** Bone volume fraction (BV/TV, **A**), trabecular thickness (Tb.Th,), structure model index (SMI, **C**), trabecular number (Tb.N, **D**), bone surface density (BS/TV, **E**), and trabecular separation (Tb.Sp, **F**) were measured for vertebrae Th13 (Th), L1 (L), and the right femora (FE) by means of micro-CT. *p ≤ 0.05. Data presented as box plots with the median indicated by solid line within the box, percentile 25 and 75 as bottom and top of the box, and percentile 0 and 100 as whiskers. Small circles represent data beyond 3x SD.

**Figure 3:**
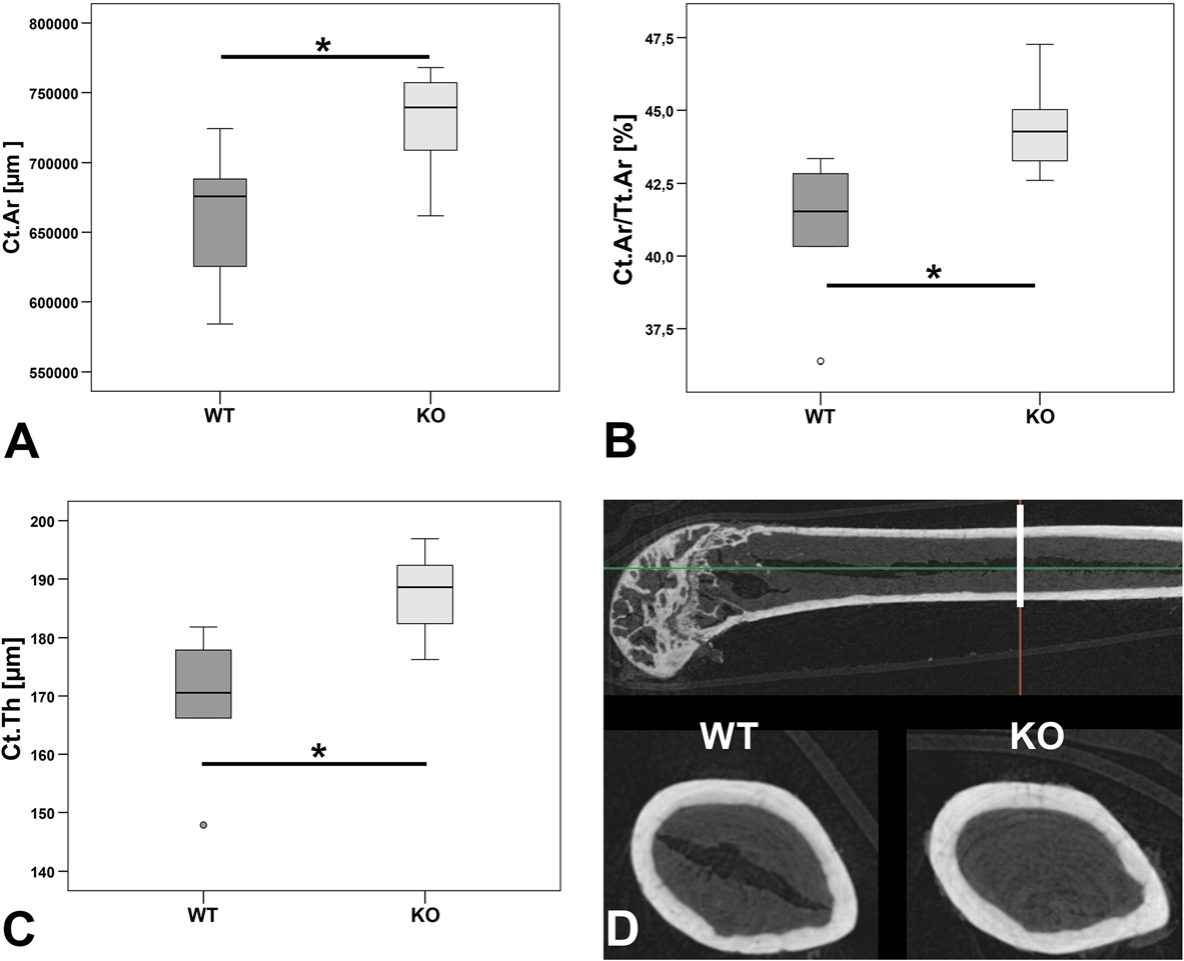
**Cortical bone parameters.** Cortical area (Ct.Ar, **A**), relative cortical bone area to tissue area (Ct.Ar/Tt.Ar, **B**), and average cortical thickness (Ct.Th, **C**) were obtained from 200 axial slices of the mid-diaphyseal area of right femora **(D)**. Data presented as box plots with the median indicated by solid line within the box, percentile 25 and 75 as bottom and top of the box, and percentile 0 and 100 as whiskers. *p ≤ 0.05. Small circles represent data beyond 3x SD.

**Figure 4:**
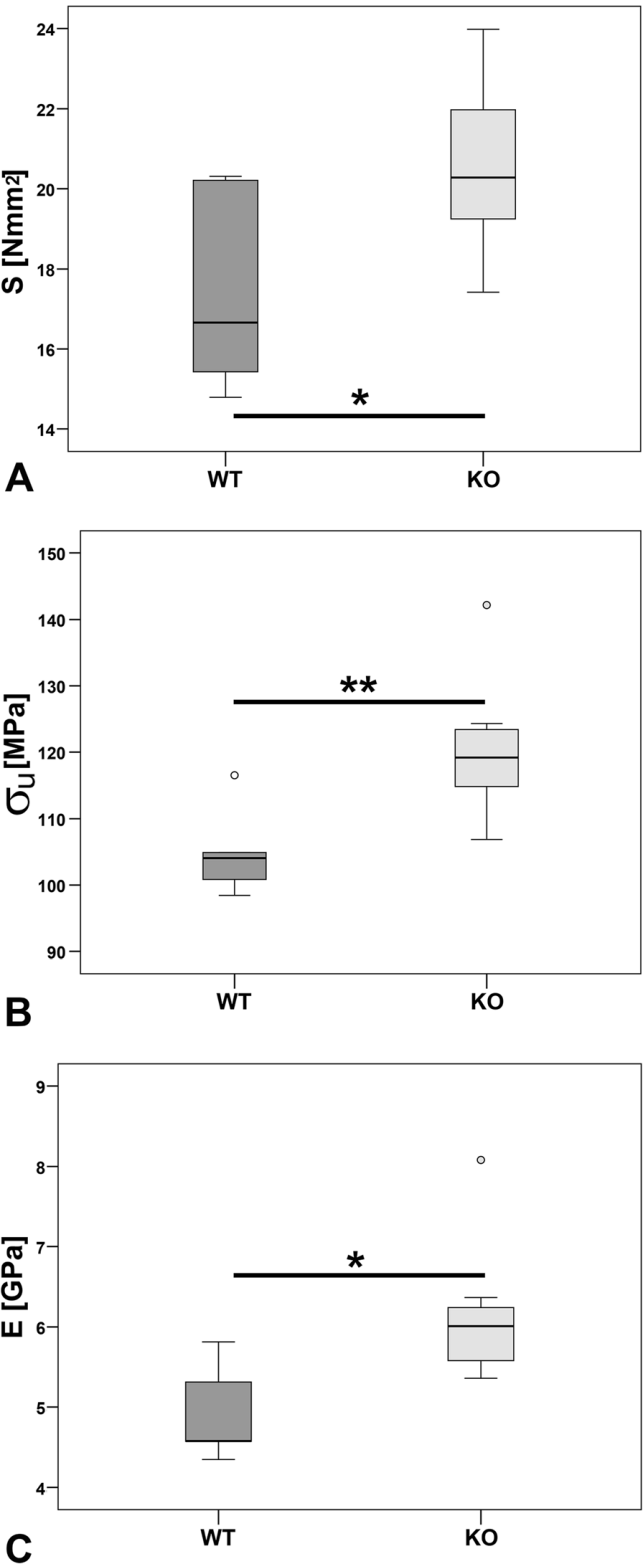
**Biomechanical bone strength.** Bending stiffness S in (Nmm^2^, **A**), maximal strain (σ_U_ in MPa, **B**), and elasticity modulus (E in GPa, **C**) were calculated for femora of α7KO and α7WT by three-point bending test. Data presented as box plots with the median indicated by solid line within the box, percentile 25 and 75 as bottom and top of the box, and percentile 0 and 100 as whiskers. *p ≤ 0.05, **p ≤ 0.01. Small circles represent data beyond 3x SD.

DXA scans did not show any significant differences in BMD (Figure 1). 3D Micro-CT analyses of the trabecular region of Th13, L1 and femur did not determine a clear result. No differences were measured for Th13 and femur whereas L1 showed an increase in BV/TV (p < 0.05, Figure 2A) and Tb.Th (p < 0.05, Figure 2B) and a reduction in SMI (p = 0.056, Figure 2C) of α7KO. No differences were detected for Tb.N (p > 0.05, Figure 2D), BS/TV (p > 0.05, Figure 2E), and Tb.Sp (p > 0.05, Figure 2F). The SMI of α7KO reflected “plate-like” structures (SMI closer to 0 whereas α7WT indicated “rod-like” structures, SMI closer to 3). 3D-Micro-CT analysis of the diaphysis resulted in a significant increase in cortical area (Ct.Ar, p < 0.05, Figure 3A), relative cortical bone area to tissue area (Ct.Ar/Tt.Ar, p < 0.05, Figure 3B), and average cortical thickness (Ct.Th, p < 0.05, Figure 3C). In accordance with the increase in cortical bone parameters biomechanical testing with the three-point bending test revealed a significant increase in bending stiffness (S, p < 0.05), maximal strain (σ_U_, p < 0.05), and elasticity modulus (E, p < 0.05) of female α7KO compared to α7WT (Figure 4).

### Bone marrow microvascularization

**Figure 5:**
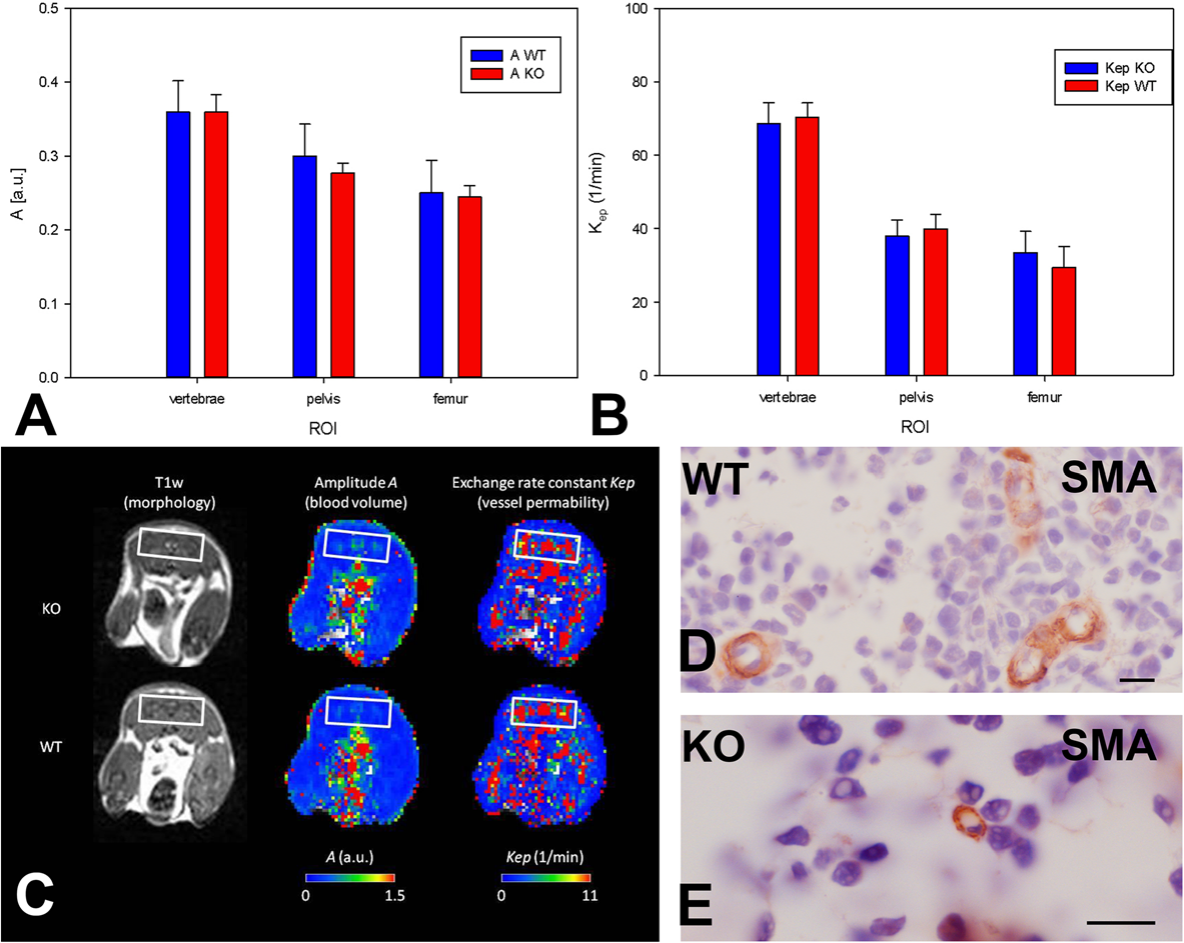
**Bone marrow vascularization.** Amplitude A **(A)** and exchange rate constant Kep **(B)** was acquired by dynamic contrast-enhanced magnetic resonance imaging (DCE-MRI; error bars: standard error) for α7KO (KO) and α7WT (WT). **(C)** Morphological images and color maps of parameters A and Kep acquired with T1 weighted morphological MRI and DCE-MRI from α7KO (upper row) and α7WT (lower row). All sections are shown in transversal orientation and contain the information of vascularization from the pelvis (white squares) as an example. The parameters are color-coded ranging from red (high values) to blue (low values). Immunohistochemical staining using a primary antibody against smooth muscle actin (SMA, red color) identified bone marrow vessels in α7WT **(D)** and α7KO **(E)** vertebrae L3. Bone marrow cells were counterstained with hematoxylin (purple). Bar: 10 μm.

Functional parameters amplitude A and exchange rate constant Kep were analyzed in the bone marrow of lumbar vertebra L5, pelvis and femur, respectively using DCE-MRI and verified with immunohistochemistry of demineralized immunolabeled sections. None of the statistical comparison performed at any anatomical site (vertebra, pelvis, femur) with DCE-MRI measurements showed significant differences between the two groups (Figure 5, Table 2). Immunolabeling were performed with antibodies detecting smooth muscle actin (SMA) and the platelet endothelial cell adhesion molecule (PECAM-1, CD31) to mark blood vessels (Figure 5D-E). Only few vessels were observed in the cortical as well as in the cancellous bone.

**Table 2:** **Quantitative DCE**-**MRI parameters**

**Group**		***A*** **[a.u.]**			***Kep*** **[1/min]**		
		**Vertebra**	**Pelvis**	**Femur**	**Vertebra**	**Pelvis**	**Femur**
**KO** **(n = 10)**	**Mean**	0.3599	0.2768	0.2446	70.3906	39.9560	29.4042
	**Median**	0.3437	0.2904	0.2579	70.7894	39.2174	24.9314
	**Std.dev**	0.0699	0.0407	0.0491	11.6716	12.3151	18.0503
	**Std.err**	0.0233	0.0136	0.0155	3.8905	3.8944	5.7080
**WT** **(n = 8)**	**Mean**	0.3603	0.3002	0.2507	68.6066	37.9580	33.4469
	**Median**	0.3291	0.2690	0.1958	68.7567	39.7596	31.3584
	**Std.dev**	0.1013	0.1223	0.1244	13.9386	12.3716	16.3246
	**Std.err**	0.0413	0.0433	0.0440	5.6904	4.3740	5.7716

KO: mice with gene deficient of α7-nAChR, WT: corresponding wild-type mice.

### Resorption capacity

**Figure 6:**
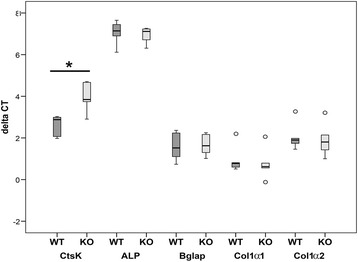
**Real**-**time RT**-**PCR.** Expression of the osteoclast marker cathepsin K (CtsK), and osteoblast markers alkaline phosphatase (ALP), osteocalcin (Bglap), collagen 1α1 (Col1α1), collagen 1α2 (Col1α2) were measured for α7WT (WT) and α7KO (KO) by real-time RT-PCR (A). Data presented as box plots with the median indicated by solid line within the box, percentile 25 and 75 as bottom and top of the box, and percentile 0 and 100 as whiskers. *p ≤ 0.05. Small circles represent data beyond 3x SD.

**Figure 7:**
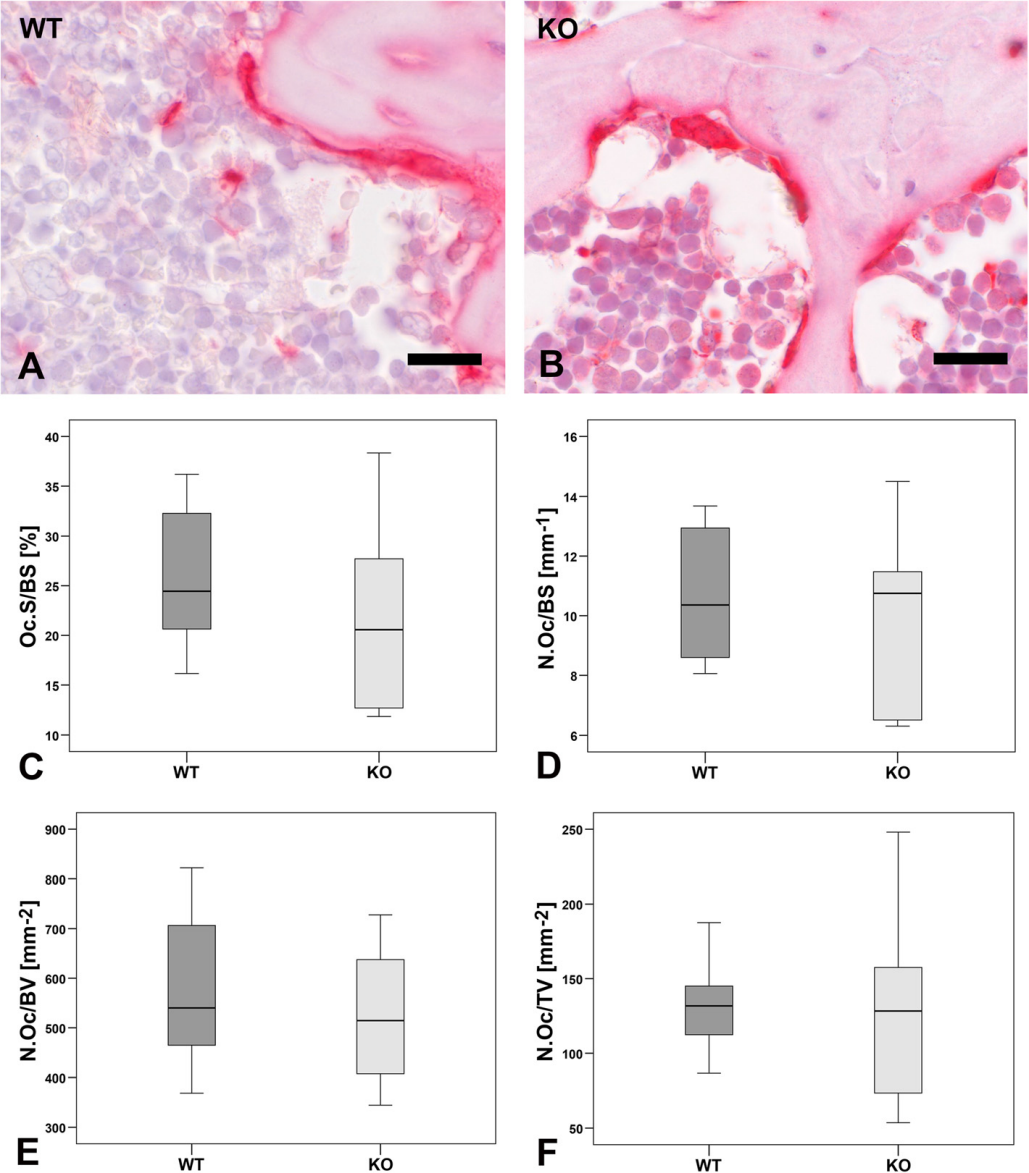
**Osteoclast histomorphometry.** Osteoclasts were determined by enzyme histochemical labeling of tartrate resistant acidic phosphatase (TRAP, **A**-**B**). The parameters osteoclast surface to bone surface (Oc.S/BS, **C**), osteoclast number to bone surface (N.Oc/BS, **D**), osteoclast number to bone volume (N.Oc/BV, **E**), and osteoclast number to tissue volume (N.Oc/TV, **F**) of α7KO (KO) compared to α7WT (WT) were calculated. Data presented as box plots with the median indicated by solid line within the box, percentile 25 and 75 as bottom and top of the box, and percentile 0 and 100 as whiskers. Small circles represent data beyond 3x SD. Bar: 20 μm.

Bone resorption was evaluated by real-time RT-PCR analysis using primers for the osteoclast marker cathepsin K that showed a significant decrease in α7KO (59%, p < 0.05, Figure 6). TRAP enzyme histochemistry was used for labeling of osteoclasts, mono- and multinucleated macrophages. Histomorphometric osteoclast parameters were determined according to [21,22]. No significant differences were measured for Oc.S/BS (p > 0.05), N.Oc/BS (p > 0.05), N.Oc/BV (p > 0.05); N.Oc/TV (p > 0.05) (Figure 7).

### Analysis of bone formation

**Figure 8:**
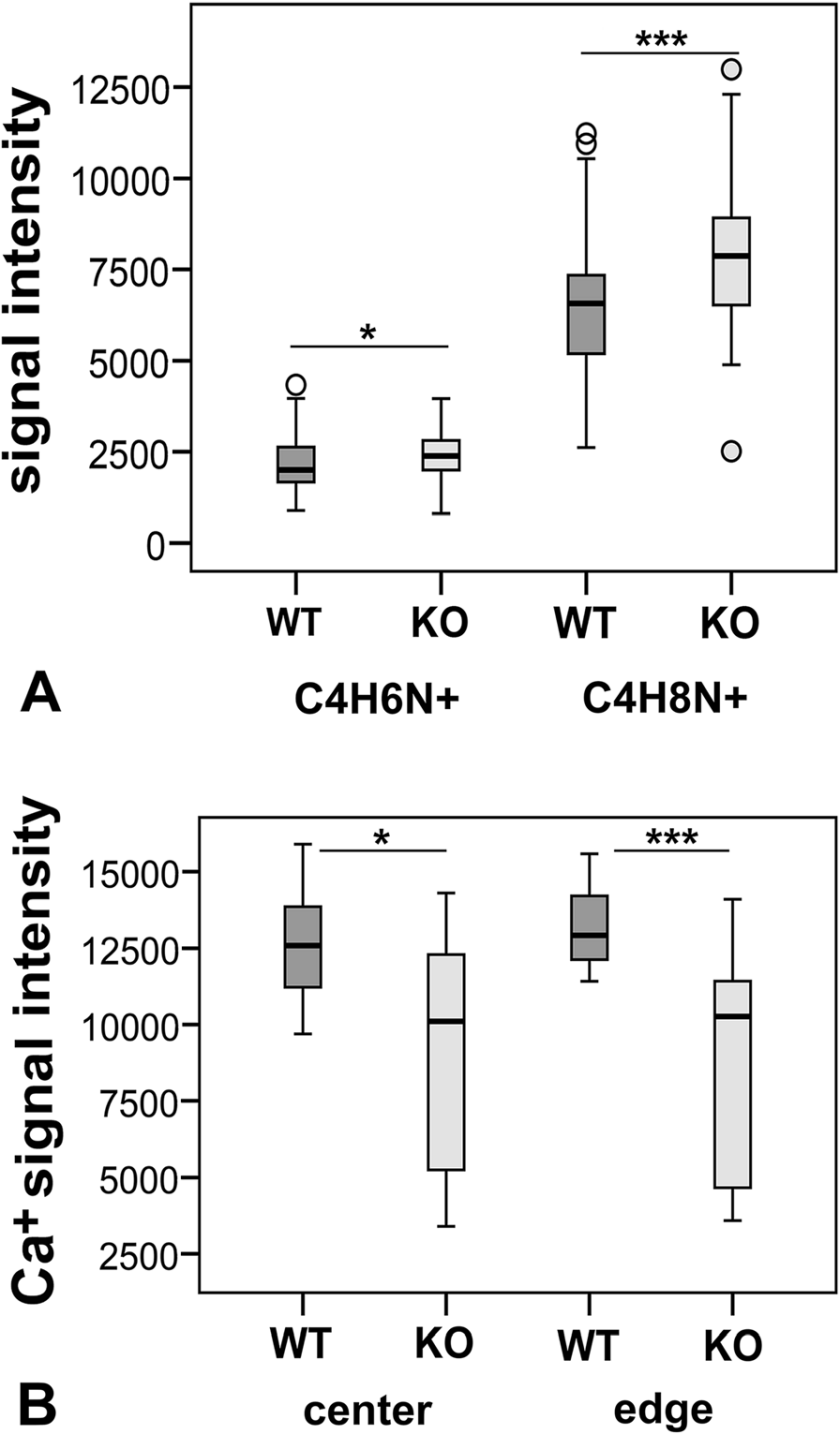
**ToF-SIMS analyses.** Mass signal intensity of **(A)** proline fragments (C_4_H_6_N^+^ and C_4_H_8_N^+^, n = 54 data evaluation areas, 9 per animal) and **(B)** calcium ion content (n = 12 data evaluation areas) of α7KO compared to α7WT. Data presented as box plots with the median indicated by solid line within the box, percentile 25 and 75 as bottom and top of the box, and percentile 0 and 100 as whiskers. Small circles represent data beyond 3 x SD. *p ≤ 0.05, ***p < 0.001.

**Figure 9:**
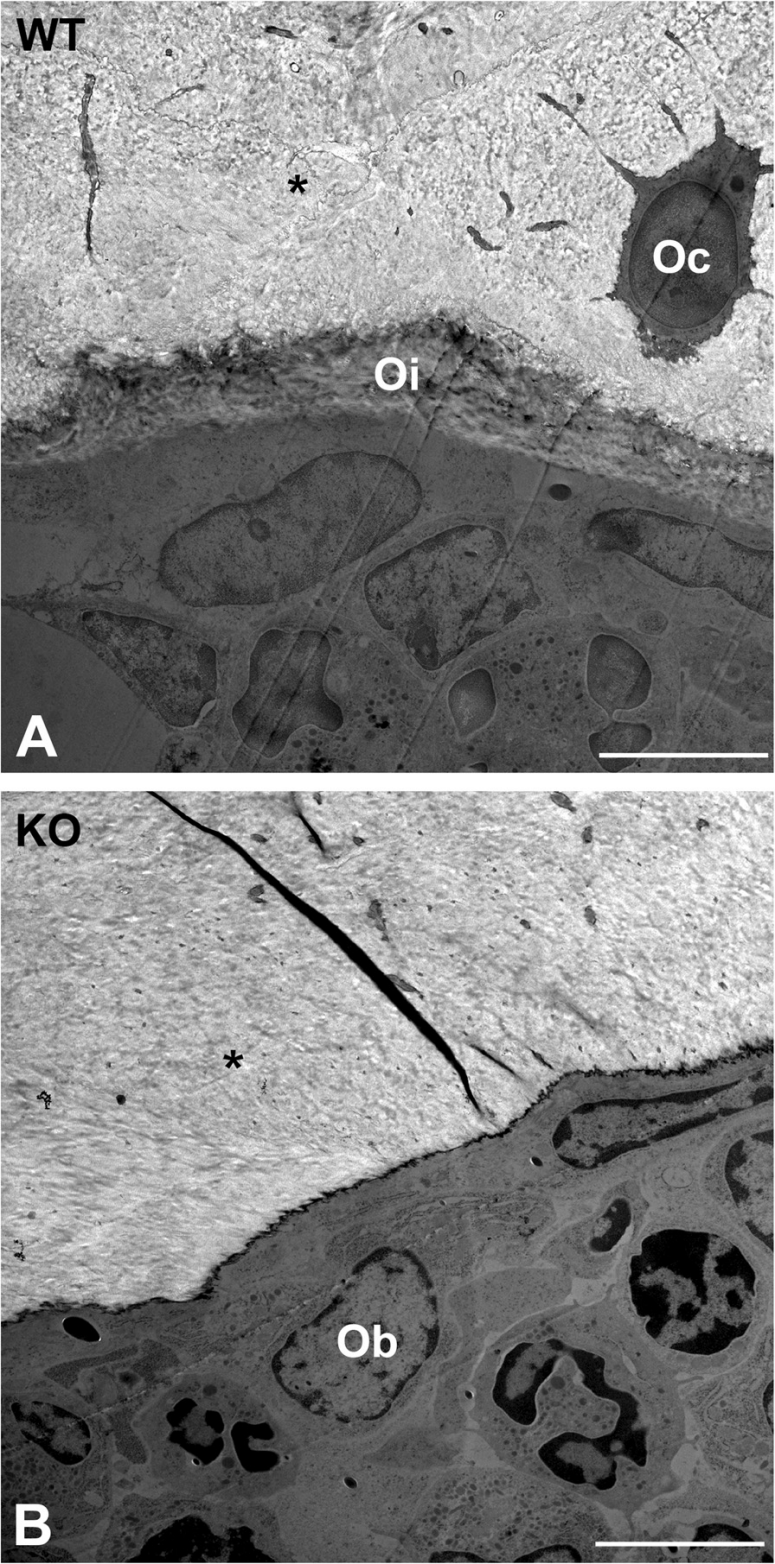
**Transmission electron microscopy.** Ultrastructure of vertebrae L4 of α7WT **(A)** and α7KO **(B)**. Oi: osteoid, Oc: osteocyte, Ob: osteoblast. Bar: 5 μm.

On the molecular level no significant differences in bone formation were determined. Real-time RT-PCR revealed no regulation of mRNA expression for the osteoblast markers alkaline phosphatase (ALP), osteocalcin (OC), Collagen 1α1 (Col1α1), and Collagen 1α2 (Col1α2) (Figure 6). ToF-SIMS analysis revealed a significant decrease in calcium ion content of trabecular center (p < 0.05) and trabecular edge (p < 0.001) in α7KO compared to α7WT (Figure 8). Combining edge and center of trabeculae a significant increase of amnio acid proline fragments in α7KO (C_4_H_6_N^+^ p < 0.05 and C_4_H_8_N^+^ p < 0.001) was measured (Figure 8). Using ultra-structural analysis by means of TEM no properly formed osteoid was found in α7KO (Figure 9).

## Discussion

In the current study we investigated bone strength, microarchitecture and composition of 16-week-old female α7KO compared to corresponding wild-type mice. We demonstrated increased biomechanical strength (e.g. bending stiffness), alterations regarding some Micro-CT parameters of vertebrae L1, up-regulation of cortical parameters (e.g. cortical thickness) by means of Micro-CT, reduced mRNA expression of the osteoclast enzyme cathepsin K, decreased trabecular calcium ion content and increased amounts of collagen fragments by means of ToF-SIMS, and no osteoid by means of TEM. Recently we investigated bones of male α7KO mice compared to α7WT by means of Micro-CT, biomechanical three-point bending test, and real-time RT-PCR [10]. Investigations of male bone did not result in significant differences between α7KO and α7WT [10]. Changes in bone mass were reported for gene deficient mice of mAChR M3 [9,10], nAChR subunit α2 [5], and male α7β2-nAChR (α7β2KO) [23]. Thus, it is supposed that AChRs are involved in bone mass regulation. Bone loss is often originated by an imbalance in bone remodelling where osteoclast bone degradation is excessive for osteoblast bone formation leading to bone disorders e.g. osteoporosis [12]. Since the strongest risk factor for postmenopausal osteoporosis is estrogen deficiency [12] we asked whether female 16-week-old α7KO hold alterations of bone characteristics. Our current results on female bone of α7KO did not demonstrate any sign of osteoporotic bone loss. On the contrary biomechanical three-point-bending test resulted in an up-regulation in bending stiffness of α7KO compared to α7WT. In line with these results we measured an increased mid-shaft cortical thickness of the female α7KO compared to α7WT by means of Micro-CT. Male α7KO did not reveal significant differences in biomechanical bone strength [10] whereas cortical thickness was not determined in our previous study. Thus, the current study showed indeed difference in bone strength of female α7KO compared to α7WT whereas male αKO did not reveal alterations.

Measurement of biomechanical parameters is less common in clinical osteoporosis diagnosis whereas determination of BMD by means of DXA scan is most frequent. A decrease in BMD standard deviation of more than 2.5 below the adult mean value is taken as sign for osteoporosis [12]. Rodents with experimental bone loss provoked by e.g. ovariectomy showed a decline in BMD [24]. Regarding female α7KO we could not determine an alteration in BMD. DXA scans for male α7KO were not performed in our previous study [10]. In male 6-month-old α7β2KO a significant increase of BMD was measured compared to wild-type mice [23]. Additionally, α7β2KO presented an increased lean body mass, decreased size of adipocytes and glycemia whereas body weight, insulin secretion and sensitivity remained unaltered [23]. The metabolic effects could be originated by hyperactivity as described for central β2-nAChR depletion [23,25]. Future analysis of α7β2KO bone microarchitecture and composition will facilitate new insights in the role of nAChR in bone.

Application of nicotine that is an exogenous agonist of nAChR induced osteoblast cell proliferation [6,26], increased gene expression of OC, Col1α1 and ALP in a human osteosarcoma cell line [26] whereas the expression of ALP in a murine osteoblast cell line was reduced [6]. We presently report that gene expression of osteoblast markers were not changed in female α7KO compared to α7WT. This is in accordance with results of our previous study on male α7KO where we also did not measure changes in Col1α1 and Col1α2 gene expression [10].

In addition to real-time RT-PCR analysis of osteoblast markers we analyzed bone formation activity of α7KO by means of ToF-SIMS measurement that was recently proved for the investigation of bone composition [15]. Using ToF-SIMS an increase of proline fragment intensity (C_4_H_6_N^+^ and C_4_H_8_N^+^) was detected for α7KO compared to α7WT. C_4_H_6_N^+^ and its derivative C_4_H_8_N^+^ are assigned to be fragments of the amino acid proline and therefore parts of collagen type 1 as already well demonstrated by means of ToF-SIMS [16]. The up-regulation of collagen synthesis might also provide an explanation for the increased biomechanical bending stiffness and cortical thickness of female α7KO. Results on bone collagen synthesis are contrary to skin collagen synthesis where Arredondo et al. (2003) described a significant down-regulation of Col1α1 in α7KO [11]. With respect to skin and bone the discrepancy in collagen synthesis might be explained by its generation by fibroblast in skin and osteoblasts in bone, respectively.

ToF-SIMS analysis was also used to determine trabecular calcium ion content that was identified by characteristic mass signals [15]. We observed a decrease of calcium content in trabecular center and trabecular edge of α7KO in comparison to α7WT. Mouse trabeculae are slim thereby the center is a region where differentiated lamellar bone is found and the edge presents the region with woven bone and osteoid. Our result on female α7KO leads us to the assumption that α7KO trabecular edge consists of lamellar bone and that these lamellar bone has reduced calcium content. However, this is highly speculative and needs to be further analyzed in future studies. In addition it has to be concerned that small effects might be missed because of the low number of analyzed animals, possible population variability, and the eventuality that while eliminating α7-nAChR gene other nAChR might be up-regulated that might be able to take over functions of α7-nAChR. General expression analysis of α7KO compared α7WT would be of high interest for bone as well as for other organs.

Bone remodeling depends on osteoblast as well as osteoclast activity. Therefore we investigated currently gene expression of osteoclast marker cathepsin K that degrades collagen type 1 and is therefore an important enzyme during bone resorption [27]. In female α7KO cathepsin K gene expression is significant down-regulated. Thus, it can be speculated that less osteoclasts are generated or that generation of degrading enzymes in osteoclasts is down-regulated in α7KO. Since nicotine is known to stimulate osteoclast differentiation and bone degradation [28] the reduction of nicotinic receptors in α7KO might provoke the down-regulation of cathepsin K expression. In male α7KO we did not investigate cathepsin K gene expression in our previous study [10]. In addition to gene expression analysis osteoclast activity can be studied by e.g. TRAP enzyme histochemistry and subsequent histomorphometry. Our results showed no significant changes in the osteoclast histomorphometrical results of female α7KO compared to α7WT. Histomorphometrical analyses have the limitation that they are done on slices which borders the method to 2-deminsions. Therefore, we performed additional 3D Micro-CT analysis as we already did in our previous study with male α7KO [10]. The balance of bone formation and degradation is described by parameters as BV/TV, BS/TV, Tb.Th, Tb.Sp, and SMI. In bone loss this parameters are demoted except SMI which is increased. In female α7KO a decrease of SMI but an increase of BV/TV and Tb.Th was measured for vertebrae L1 whereas analysis of vertebrae Th13 and femur did not result in significant differences. All three analyzed bones are well attributed to human osteoporosis [29]. Thus, in addition to biomechanical testing, determination of BMD and ToF-SIMS analysis also Micro-CT investigation of trabecular bone does not show any evidence of bone loss in α7KO. In contrast to α7KO significant bone loss was determined in mice with deficiency of nAChR subunit α2 [5] and mAChR M3 [9,10] whereas double knockout of α7β2nAChR resulted in significant bone mass accrual [23].

Besides, the non-neuronal presence of α7-nAChR and its supposed impact in bone remodeling it was proven that bone is under neuronal control [5,30]. Blood flow of the common carotid artery is increased by nicotine stimulation through α7-nAChR in cat medulla [31,32]. In addition to the central nervous system also the autonomic nervous system controls blood flow. In α7KO a decreased baroreflex-mediated tachycardia was detected indicating that α7-nAChR participate in the autonomic reflex that maintains blood pressure homeostasis [33]. Blood circulation of bone is also regulated via the autonomic nervous system. Stimulation of sympathetic nerves decreased bone blood flow [34] and diminished bone mass accrual [35]. However, current study did not reveal any regulation in bone blood flow through α7-nAChR because no significant change was observed in bony blood flow of α7KO.

## Conclusions

In the present study, we identified differences in bone strength, composition and microarchitecture of female α7KO. Our results showed that deficiency α7-nAChR did not induce bone loss in contrast to nAChR subunit α2 [5] and mAChR M3 [9,10]. Similar to male mice with gene deficiency of α7β2-nAChR [23] female α7KO revealed an increase in bone mass. However, the presently detected bone mass accrual could not be detected in male α7KO as determined previously [10]. Thus, bone strength and composition are different in female and male α7KO. Further studies are necessary to determine gender specific effect of α7-nAChR in bone homeostasis.
